# Response to the Letter to the Editor: ʻLife expectancy of UK physicians in the early 21st century: An analysis of 1,000 fellows from the Royal College of Physicians’ Munk’s Roll’

**DOI:** 10.1016/j.clinme.2026.100620

**Published:** 2026-07-03

**Authors:** I. Dafydd Woolley, Peter N. Taylor, Justyna Witczak, Fizzah Iqbal, Anna Scholz, Andrew Lansdown

**Affiliations:** Cardiff University School of Medicine, Cardiff, UK; Thyroid Research Group, Systems Immunity Research Institute, Cardiff University School of Medicine, Cardiff, UK; Department of Immunology and Immunotherapy, School of Infection, Inflammation and Immunology, College of Medicine and Health, University of Birmingham, UK; Department of Endocrinology, University Hospital of Wales, Heath Park, Cardiff, UK

We thank Dr Williams for her thoughtful comments regarding our recent analysis of 1,000 fellows from the Royal College of Physicians' Munk's Roll.

We agree, as indeed we indicate, that observational studies such as ours cannot establish the reasons underlying differences in longevity and that caution is required when interpreting potential explanatory mechanisms. We also agree that the associations reported in our study should not be interpreted as evidence of causality. However, we would respectfully note that the discussion in our article was informed by the available literature and was presented as a series of possible explanations rather than definitive conclusions.

In relation to sex differences, our discussion was informed by several strands of published evidence. We cited the recent study by Patel *et al*.,^1^ which examined mortality among more than 3.6 million US healthcare workers and demonstrated that female physicians did not exhibit the mortality advantage generally observed among women in other occupations, with Black female physicians experiencing the highest mortality among physician subgroups. We also cited studies describing higher rates of discrimination, abuse and burnout during medical training,^2^ greater domestic and parenting responsibilities among women physician-researchers,^3^ and the recognised health consequences of physician burnout.^4^ While these studies derive from contemporary populations, there is a paucity of equivalent data for the historical physician cohorts represented within Munk's Roll. Consequently, contemporary physician studies provide the closest available evidence base from which hypotheses may be generated regarding the mechanisms underlying our observations.

Importantly, our intention was not to suggest that these factors had been proven to explain the differences observed in our cohort, but rather that they represent plausible mechanisms worthy of further investigation. Indeed, we share Dr Williams' view that understanding why female physicians in our cohort demonstrated lower longevity than their male counterparts remains an important unanswered question. We would also note that the observation reported by Patel *et al*. that female physicians, and particularly Black female physicians, experience unexpectedly adverse mortality outcomes suggests that some of the patterns observed in historical physician cohorts may still be relevant today.^1^

Similarly, we agree that the relationship between migration, ethnicity, country of qualification and longevity is complex. We cited the healthy migrant effect as one possible explanation for the longer lifespans observed among some internationally trained physicians, while acknowledging that the mechanisms underlying these differences remain uncertain. We welcome the additional literature highlighted by Dr Williams. However, it is worth noting that the cited study examined mortality patterns in the general South Asian migrant population rather than physicians.^5^ Interestingly, the study reported findings in the opposite direction to those observed in our physician cohort, with evidence of a mortality advantage among South Asian migrants. We believe this discrepancy further emphasises the need for additional research into the complex relationships between migration, ethnicity, professional background and longevity, rather than providing a direct explanation for the findings observed in Munk's Roll.

More broadly, we believe the correspondence highlights the limitations inherent in historical biographical datasets. Munk's Roll contains rich information on physicians' careers and lives but relatively little information regarding ethnicity, socioeconomic status, workload, discrimination, migration experiences and other factors that may influence mortality. To our knowledge, there are very limited data examining mortality patterns among historical physician cohorts stratified by country of qualification. Consequently, there remains a striking paucity of evidence regarding the determinants of longevity among historical physician populations.

Since publication of our study, we have explored methods for large-scale electronic extraction and analysis of data from Munk's Roll. This work has enabled the development of substantially larger datasets than were previously feasible through manual extraction alone. We hope that these approaches will allow future studies to investigate historical trends in physician longevity, training, migration and workforce demographics at a scale capable of addressing many of the important questions raised by both our article and Dr Williams' correspondence.

We are grateful to Dr Williams for highlighting these important issues. We hope that this discussion encourages further research into physician wellbeing, inequality and longevity. While the mechanisms remain uncertain, the observations reported in our study remain robust and, we believe, continue to provide valuable insights into the lives of physicians recorded in Munk's Roll.

## CRediT authorship contribution statement

**Justyna Witczak:** Writing – review & editing. **Peter N. Taylor:** Writing – original draft. **I. Dafydd Woolley:** Writing – review & editing. **Andrew Lansdown:** Writing – review & editing. **Anna Scholz:** Writing – review & editing. **Fizzah Iqbal:** Writing – review & editing.

## Funding

This research did not receive any specific grant from funding agencies in the public, commercial, or not-for-profit sectors.

## Declaration of competing interest

The authors declare that they have no known competing financial interests or personal relationships that could have appeared to influence the work reported in this paper.

## References

[1] Patel V.R., Liu M., Worsham C.M., Stanford F.C., Ganguli I., Jena A.B. (2025). Mortality among US physicians and other health care workers. JAMA Intern Med.

[2] Hu Y.Y., Ellis R.J., Hewitt D.B. (2019). Discrimination, abuse, harassment, and burnout in surgical residency training. N Engl J Med.

[3] Jolly S., Griffith K.A., DeCastro R., Stewart A., Ubel P., Jagsi R. (2014). Gender differences in time spent on parenting and domestic responsibilities by high-achieving young physician-researchers. Ann Intern Med.

[4] West C.P., Dyrbye L.N., Shanafelt T.D. (2018). Physician burnout: contributors, consequences and solutions. J Intern Med.

[5] Hayes L., White M., McNally R.J.Q., Unwin N., Tran A., Bhopal R. (2017). Do cardiometabolic, behavioural and socioeconomic factors explain the “healthy migrant effect” in the UK? Linked mortality follow-up of South Asians compared with white Europeans in the Newcastle Heart Project. J Epidemiol Community Health.

